# Quorum Sensing Bacteria in the Phycosphere of HAB Microalgae and Their Ecological Functions Related to Cross-Kingdom Interactions

**DOI:** 10.3390/ijerph19010163

**Published:** 2021-12-24

**Authors:** Yanchao Zhang, Li Zheng, Shuai Wang, Yangguo Zhao, Xiyuan Xu, Bin Han, Tianyi Hu

**Affiliations:** 1 College of Environmental Science and Engineering, Ocean University of China, Qingdao 266100, China; zhangyanchao@stu.ouc.edu.cn (Y.Z.); ygzhao@ouc.edu.cn (Y.Z.); 2 Key Laboratory of Marine Ecological Environment Science and Technology, First Institute of Oceanography, Ministry of Natural Resources, Qingdao 266061, China; wangshuai@fio.org.cn (S.W.); xuxiyuan@issas.ac.cn (X.X.); happy568888@163.com (T.H.); 3 Qingdao National Laboratory of Marine Science and Technology Pilot, Functional Laboratory of Marine Ecology and Environmental Science, Qingdao 266071, China; hanbin@fio.org.cn

**Keywords:** AHL, DMSP, film formation, HABs, quorum sensing

## Abstract

It has been proven that the relationship between microalgae and bacteria affects the dynamic process of harmful algal blooms (HABs). Microalgae-associated microorganisms widely exist in the phycosphere and play an essential role in algae-bacteria cross-kingdom interactions. Among these processes, quorum sensing (QS), as a communication system of bacteria, is thought to participate in algae-bacteria interactions. However, the species of QS bacteria in the phycosphere and their ecological function are still unknown. In this study, microalgae-associated microorganisms with a QS system were screened by the biosensor method and identified based on 16S rRNA gene analysis. The types and number of acyl-L-homoserine lactone (AHL) signalling molecules produced by QS bacteria were analysed by thin layer chromatography (TLC) bioautography and gas chromatography-mass spectrometer (GC-MS). The film formation, β-dimethylmercaptopropionic (DMSP) degradation and algae growth effects of QS bacteria were investigated. The results showed that 113 QS bacteria were isolated from 842 microalgae-associated bacteria. Detection of AHL molecules in 10 different species of QS bacteria showed that most of them were *N*-(3-Oxodecanoyl)-L-homoserine lactone (OC10-HSL), *N*-Octanoyl-L-homoserine lactone (C8-HSL) and *N*-(3-Oxooctanoyl)-L-homoserine lactone (OC8-HSL). All 10 QS bacteria had film-forming ability, and they could degrade DMSP (except strain E26). The crude metabolic extracts of the 10 QS bacteria can inhibit or promote microalgae growth to different degrees. Our study is helpful to understand the role of microalgae-associated microorganisms with the QS system in algae-bacteria interactions and community succession of HAB microalgae.

## 1. Introduction

During the process of growth, microalgae release dissolved organic matter (DOM) and particulate organic matter (POM) into the environment. A unique microenvironment is formed around algal cells called the phycosphere [1,2]. Microalgae-associated microorganisms widely exist in the phycosphere and play an essential role in algae-bacterial cross-kingdom interactions [3,4,5]. Previous studies found that the bacteria in the phycosphere proliferated by utilizing organic material released by microalgae [5]. In turn, some of them could provide a large amount of nutrients, such as vitamins, siderophores, and auxins, to promote the growth of the microalgae [6,7,8]. Others secrete algicidal substances that inhibit the growth of microalgae or lead to the aggregation and deposition of microalgae [9,10]. This process is thought to be a mechanism used to control the numbers and populations of microalgae to maintain the ecological balance of the phycosphere [5]. Evidence from field investigations has shown that microalgae-associated microorganisms play a role in regulating the population size of red tide algae during the development of red tides [11,12]. For example, a field investigation found that the number of environmental microorganisms gradually increased during the extinction of a red tide. Previous researchers thought that algal microorganisms primarily played an algae-killing role [13,14].

Considering that the flora has certain social attributes, its ecological function is not dependent on the individual, but on the group. As early as the 1990s, studies confirmed the existence of information exchange between bacteria via a QS system [13]. After that, a large number of studies found that marine microorganisms could use AHLs as a specific communication language to regulate their population density and exert corresponding biological effects [14]. We speculate that bacteria regulate some functions (biofilm formation, DMSP degradation, etc.) through AHLs, thus influencing the interaction between microalgae and bacteria in the phycosphere.

In the phycosphere, there is a close and complex interaction between microalgae and bacteria. For example, microalgae, as a primary producer, provide nutrients for bacteria and stimulate the proliferation of bacteria by secreting substances (such as [^3^H] thymidine), which can enhance the bacterial ability to form biofilms; microalgae can also kill or repel some bacteria by producing antibacterial substances, such as fatty acids, polyunsaturated aldehydes and halogenated compounds [15,16,17]. Bacteria can produce signalling molecules called autoinducers (AIs) during the growth process. When the bacterial density reaches a certain concentration threshold, the AI will combine with the AI receptor and activate the expression of functional genes, causing the bacteria to exhibit unique physiological and biochemical phenomena [18].

AHLs are the most widely studied QS signalling molecules in Gram-negative bacteria [19]. The most basic structure of this type of signal molecule is comprised of an acyl chain and a homoserine lactone ring, which can be detected by a variety of methods, such as the bacterial biosensor method, TLC-bioautography, GC-MS and liquid-phase mass spectrometry technology (LC-MS) [14,20,21,22,23].

Studies have shown that the bacterial QS system mediated by AHLs can be used as a communication language between bacterial communities to participate in the regulation of various ecological functions, such as the degradation of DMSP, the formation of biofilms, microalgae growth inhibition and the production of antibiotics [24,25,26]. Because microalgae and bacteria can engage in cross-kingdom interactions through QS, QS bacteria are one of the important types of bacteria in the phycosphere. In addition, studies have shown that most marine QS bacteria belong to the *Roseobacter* group, which indicates that QS bacteria may occupy an essential ecological position in the relationship between algae and bacteria [27,28,29]. Lv et al. showed that QS bacteria are involved in the regulation of “microalgae-bacteria” and “bacteria-bacteria” during HABs [30]. Huang et al. explained the microbial behaviour mediated by the QS signal from the perspective of chemical ecology, showing that it regulates a variety of biological functions, such as the niche construction, biofilm formation, matter metabolism (C, N, S and Fe) and algicidal character [31]. However, there is still a significant lack of understanding of the species of microalgae commensal QS bacteria, the signal molecules that they produce, and their biological functions. Xu and Chi conducted preliminary studies on algae inhibitory activity, but systematic research is lacking [32,33].

In this study, we isolated and screened AHL classification QS bacteria from microalgae, analysed the types of their signalling molecules, and studied their possible ecological functions in the relationship between microalgae and bacteria, including the ability to form biofilms, degrade DMSP and affect the growth of microalgae. The purpose of this paper is 1. to identify the diversity of QS bacteria and signalling molecules they produce and 2. to understand the ecological role of these QS bacteria in the relationship between algae and bacteria. Additionally, focusing on the study of microbial behaviour mediated by QS signals will probably provide a new perspective on the microecological processes of algal blooms.

## 2. Materials and Methods

### 2.1. Sample Source and Pretreatment of Microalgae

The eight species of HAB microalgae used in this experiment were provided by the Institute of Oceanology, Chinese Academy of Sciences: *Prorocentrum minimum*, *Alexandrium tamarense*, *Chattonella marina*, *Prorocentrum donghaiense*, *Prorocentrum lima*, *Heterosigma akashiwo*, *Alexandrium streptosus*, and *Karenia mikimotoi*.

Two millilitres of microalgae in the exponential growth phase was centrifuged at 7000× *g* rpm/min for 2 min. The microalgae pellet was washed with sterile seawater 3 times to remove unattached microorganisms. Then, the microalgae were resuspended in 1 mL sterile seawater and ground in a 1 mL tissue grinder (Wheaton, IL, USA).

### 2.2. Isolation and Purification of Microalgae Associated Bacteria

The ground microalgae suspension was serially diluted with sterile seawater to 10^−2^, 10^−3^, 10^−4^, and 10^−5^, and 200 μL of each dilution was applied onto 2216E agar plates in triplicate. The plates were incubated at 25 °C, and colonies with different colony sizes, colours and morphologies were picked for further purification after two weeks of cultivation.

### 2.3. Screening of Quorum Sensing Bacteria

All purified microalgae-associated bacteria were spotted onto 2216E agar plates and grown for three days at 25 °C until colonies or patches formed. The QS bacteria were tested by diffusion agar plate assays using the reporter *Agrobacterium tumefaciens* (pJZ372) (pJZ384) (pJZ410) KYC55-based biosensor system. This reporter strain is a sensitive strain to detect AHLs with carbon chain lengths between C4 and C14. First, 90 mL of sterile and fully molten soft agar (0.8%) was supplemented with 5 mL of 20 × AT salts (15 mmol/L (NH4)_2_SO_4_, 0.6 mmol/L MgSO_4_·7H_2_O, 0.06 mmol/L CaCl_2_·2H_2_O and 0.0071 mmol/L MnSO_4_·H_2_O), 5 mL of 20 × AT buffer (79 mmol/L KH_2_PO_4_, pH = 7.0), and 1 mL of 50% (*w*/*w*) glucose. Then, 200 μL of 20 mg/mL X-gal and 1 mL of KYC55 cells (OD_600nm_ = 12) were added to the soft agar at 45 °C. The mixture was poured over the colonies or patches containing plates and allowed to solidify. The plates were placed in a refrigerator at 4 °C overnight for diffusion of the AHL molecules and then incubated at 30 °C for 24 h. Positive AHL-producing strains were recorded as a visible blue colour appearing around the colony or patch.

### 2.4. Strain Identification and Phylogenetic Analysis

The QS bacteria were identified by the 16S rRNA gene sequencing method. The 16S rRNA gene was amplified by PCR using 27F and 1492R as the primers [34]. The PCR products were sequenced by Beijing Kinco Xinye Biotechnology Co., Ltd. The sequencing results were analysed by NCBI’s Nucleotide-BLAST tool for homology comparison analysis (http://www.ncbi.nlm.nlh.gov, accessed on 31 December 2019). The genetic relationship was determined according to the similarity of the sequences. Multiple sequence comparisons were performed by MEGA (version 10.0.5, MEGA team, Philadelphia, PA, USA). A phylogenetic tree was constructed using the neighbour-joining method.

### 2.5. Extraction and Identification of AHLs

The AHL signalling molecules were extracted according to the method of Chi, and the types were identified using TLC-bioautography and GC-MS methods [33]. A 200 mL culture of each QS bacterium was grown to stationary phase in 2216E liquid medium (hopebio, Qingdao, China) at 25 °C with 180 rpm/min shaking. The cultures were adjusted to pH = 7.0 with HCl (6 mol/L) and extracted with 200 mL of ethyl acetate. The ethyl acetate phase was evaporated in a rotary evaporator at 45 °C for 30 min and then blown dry with ethyl acetate via nitrogen. The dried extract was suspended in 2 mL ethyl acetate (0.01% acetic acid) for AHL analysis.

The AHL standards were *N*-Hexanoyl-L-homoserine lactone (C6-HSL), C8-HSL, *N*-(β-Ketocaproyl)-L-homoserine lactone (OC6-HSL), OC8-HSL and OC10-HSL in the lab. The AHL standards and the crude extracts of QS bacteria were sequentially sampled onto reversed-phase TLC (TLC aluminium sheets, 20 × 20 cm, silica gel 60F254, Merck, Kenilworth, NJ, USA) and layered with 60% methanol. The TLC plates were dried and placed in a large square sterile petri dish, and the AHL reporter soft agar described in Section 2.3 was poured on top of the plate. The plate was incubated at 30 °C overnight to allow the appearance of blue spots associated with the presence of AHLs. Observing the number and position of blue spots on the TLC plate, we calculated the ratio shift value (R_f_) and compared them to the AHL standard [22].

GC-MS (7980A/5975C, Agilent Technologies, Santa Clara, CA, USA) was used to analyse the types of AHL signalling molecules. The crude extract (1 mL) of QS bacteria was blown to dry and redissolved in 200 μL chromatographic pure ethyl acetate, and a 100 μg/mL AHL standard was prepared for comparison. The prepared sample (1 μL) was injected in split mode (5:1) into an HP-5MS quartz capillary column (30 m × 250 μm × 0.25 μm). High-purity helium was used as a carrier gas at a flow rate of 1 mL/min. The GC injector temperature was set at 200 °C, and the transfer line temperature was 280 °C. The heating program was set as follows: the initial temperature was 150 °C, then it increased to 280 °C at a flow rate of 25 °C/min and then was maintained for 3 min. The ion detection mode (SIM) m/z 143 was selected. The mass spectrometry conditions were as described by Chi [33].

### 2.6. The Biological Functions of QS Bacteria

#### 2.6.1. Biofilm Formation

##### Crystal Violet Staining Method

The crystal violet staining method was utilized to detect the bacteria’s film-forming ability [35]. Briefly, 10 μL QS bacterial suspension (OD_600nm_ = 0.3) and 190 μL 2216E liquid medium were added to a 96-well microplate (Corning, USA) and cultured at 25 °C for 36 h. Then, the culture medium was discarded, and the microplate was washed twice with 1 × PBS solution (Solarbio, Beijing, China). After fixing with 200 μL Bouin’s solution (LEAGENE, China) for 25 min, the biofilm was stained with 250 μL crystal violet solution (0.1%, *w*/*v*) for 30 min. After removing the unstained crystal violet solution with sterile water, the crystal violet dye adsorbed on the biofilm was dissolved with 200 μL 95% (*v*/*v*) ethanol by shaking for 20 min. The solution was used to measure the optical density value by a microplate reader at 600 nm. *E. coli* DH5α was used as the negative control, and its OD_600nm_ is presented as the ODc. OD < ODc indicated no biofilm formation ability, ODc < OD < 2*ODc indicated weak biofilm formation ability, 2*ODc < OD < 4*ODc indicated medium biofilm formation ability, and 4*ODc < OD indicated strong biofilm formation ability [35].

##### Confocal Laser Scanning Microscope Detection Method (CLSM)

A three-channel confocal laser scanning microscope (FLUOVEWFV1000, Olympus, Japan) was used to observe the morphological characteristics and formation speed of the biofilm. COMSTAT 2.0 microbial envelope analysis software was used to quantitatively analyse the biofilm tissue structure.

A sterilized slide (φ = 14 mm) was placed in a flat-bottom 24-well plate (Corming, USA), 100 μL QS bacterial solution (OD_600nm_ = 0.3) and 2 mL liquid 2216E liquid medium were added to each well, and the cells were cultured at 30 °C for 3 days. The slide was washed with 1 × PBS solution to remove free living bacteria and fixed with 4% tissue cell fixative (Solarbio, Beijing, China) for 2 h. After washing with 1×PBS solution, the slide was stained with 20 μmol/L PI (Solarbio, Beijing, China) in the dark for 25 min and washed with 1 × PBS solution. Two-dimensional observation and three-dimensional scanning of the biofilm on the slide were carried out using a three-channel confocal laser scanning microscope with an objective lens (UPlansSApo 20 × 0.75). The scanning images were processed by Olympus FV10-ASW (version 4.0) and reconstructed into 3D images in which the excitation and emission light wavelengths were 559 nm and 570–670 nm, respectively.

#### 2.6.2. DMSP Consumption

Two hundred microlitres of QS bacterial suspension in seawater (OD_600nm_ = 0.3) was mixed with 1.8 mL DMSP medium (0.6 mmol/L DMSP, 4 mmol/L NH_4_Cl, 30 nmol/L NaH_2_PO_4_, 100 nmol/L EDTA-Fe, 100 nmol/L ZnCl_2_, 100 nmol/L MnCl_2_, 1 nmol/L CoCl_2_∙6H2O, 1.186 mmol/L vitamin B_1_, 1.476 nmol/L vitamin B_12_, and 8.186 nmol/L biotin) in a sterilized gas-phase injection vial (Agilent, Santa Clara, CA, USA) and cultured at 25 °C and 200 rpm/min in the dark for 12 h. The sample was tested by headspace gas chromatography (GC, Agilent 7890B, Santa Clara, CA, USA) without pretreatment. First, Dimethyl sulfide (DMS) in the sample was collected using a headspace sampler, and its concentration was detected by gas chromatography according to Williams’ method [36]. The consumption of DMSP in the sample was reflected by the content of equimolar DMS [37].

#### 2.6.3. Detection of Microalgae Cell Density

Six typical red tide microalgae, *P. donghaiense* (PD), *K. mikimotoi* (KM), *A. tamarense* (AT), *C. marina* (CM), *P. lima* (PL), and *Phaeodactylum tricornutum* (PT), were selected as the target microalgae. The above six microalgae in the exponential growth phase were diluted to 1.0 × 10^4^ cells/mL, 2.5 × 10^3^ cells/mL, 3.0 × 10^4^ cells/mL, 2.5 × 10^3^ cells/mL, 5.0 × 10^3^ cells/mL and 3.0 × 10^3^ cells/mL with f/2 medium as the test microalgal culture. The metabolite extract of QS bacteria at 20 mg/mL was prepared with dimethyl sulfoxide (DMSO) using the ethyl acetate extract described in Section 2.5, and 2216E liquid medium was used as a negative control. Microalgae inhibition activity was tested by mixing 50 μL metabolite extract with 5 mL test microalgal culture and incubated in a light incubator at 25 °C under 76 mmol/m^2^/s illumination for 5 days. The density of the microalgae cells and the growth inhibition rate (*IR*) of each microalgae were calculated by the following formula:

(1) $$IR=\frac{\rho 0-\rho }{\rho 0}\;\times \;100\%$$

In the formula, ρ0 was the cell density of the negative control microalgae, and ρ was the cell density of the microalgae treated with the crude bacterial metabolite extract.

## 3. Results

### 3.1. Molecular Identification and Phylogenetic Tree Analysis of QS Bacteria

A total of 842 microalgae-associated bacterial strains were isolated from eight typical red tide algae. Among them, 113 strains (13.42%) showed the QS phenomenon based on a biosensor detection system. After identification of the QS strains, the repetitions were removed, and 10 different bacterial strains were retained for the following study (Table 1). Next, they were compared with the closest match in GenBank. Although it was found that the similarity of the 16S rRNA gene sequences across the 10 QS strains was very high, such as C31, G115, H52 and G74, they were not the same strain in terms of morphological distinction.

**Figure 1:**
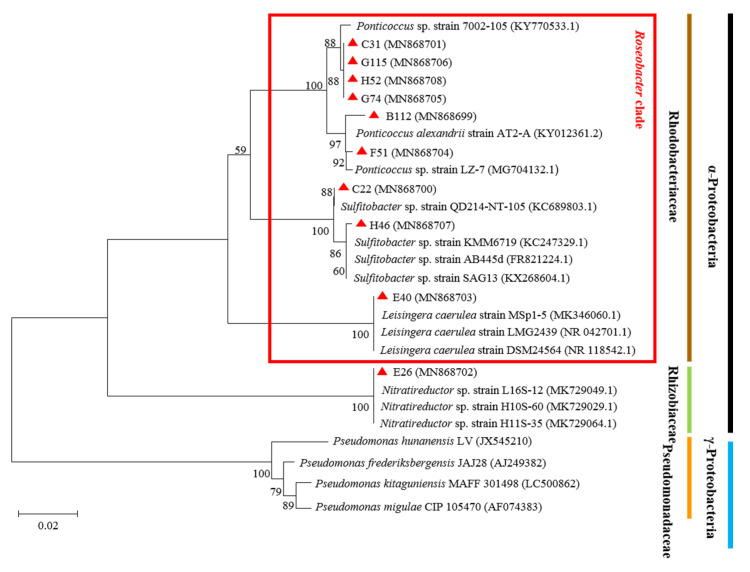
Phylogenetic tree of the QS bacteria isolated from marine HAB species. The black line represents the α-Proteobacteria; the blue line represents the γ-Proteobacteria; the brown line represents Rhodobacteriaceae; the green line represents Rhizobiaceae; the orange line represents Pseudomonadaceae; the red box represents the QS bacteria belonging to the *Roseobacter* clade; the 10 QS strains are indicated by red triangles.

According to the genetic relationship between similar strains in the GenBank database and the 16S rRNA of the QS strains, a multiple sequence alignment was performed, and a phylogenetic tree was constructed (Figure 1). The results showed that the 10 QS strains belonged to four genera, *Sulfitobacter*, *Ponticoccus*, *Leisingera* and *Nitratireductor*, which all fall in the class of α-Proteobacteria. However, most QS bacteria belonged to *Sulftobacter* (two strains) and *Ponticoccus* (six strains). Among them, nine strains of bacteria (B112, C22, C31, E40, F51, G74, G115, H46, and H52) belonged to the *Roseobacter* cluster.

### 3.2. Profile of the AHL Signalling Molecules

**Figure 2:**
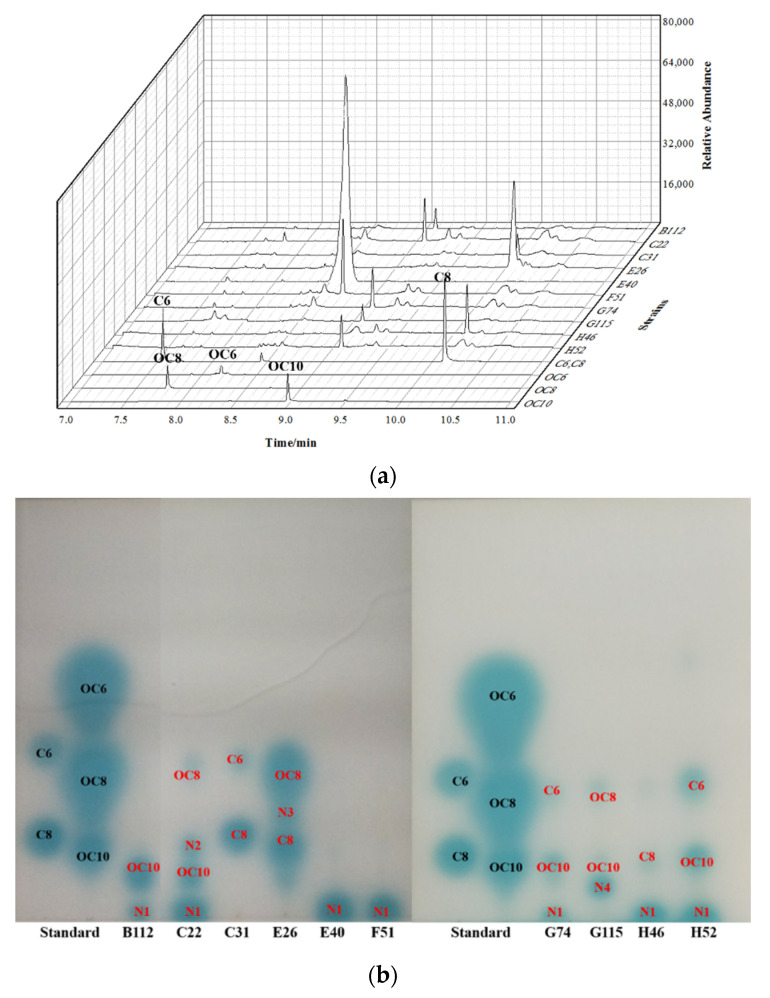
AHLs produced by microalgae-associated QS bacteria via GC-MS (**a**) and TLC (**b**) analysis. (**a**) Ethyl-acetate extracted 10 QS bacteria’ supernatant analysed by GC-MS. The standards included five AHLs: C6 (retention time: 7.54 min), C8 (retention time: 10.17 min), OC6 (retention time: 8.21 min), OC8 (retention time: 7.83 min), OC10 (retention time: 8.96 min). (**b**) Detection of AHLs signalling molecules using KYC55 as the biosensor strain. The presence of AHLs substance was visualized by TLC. The standards (C6, C8, OC6, OC8, OC10) were marked in black. The AHLs produced by 10 QS bacteria were marked in red.

TLC-bioautography and GC-MS were used to analyse the profile of AHL signalling molecules produced by the 10 QS bacteria. The results showed that the QS bacteria produced eight different AHL signalling molecules, including two OC series AHLs: OC8-HSL (R_f_ = 0.318), OC10-HSL (R_f_ = 0.139) and two C series: C6-HSL (R_f_ = 0.397), C8-HSL (R_f_ = 0.189), as well as 4 undetermined AHLs: N1–N4 (Figure 2). The most dominant type of AHL signalling molecule was N1, produced by 7 strains, followed by OC10-HSL, produced by 5 strains. Strains C22, E26 and G115 produced one detectable AHL signalling molecule. Strains B112, C22, E40, F51, G74, H46 and H52 all produced a unique signal molecule N1 with an R_f_ of almost 0. It should be a high molecular weight AHL that was not coincident with the R_f_ of the five standards. Strains B112, C22, G74, G115 and H52, which belong to the *Roseobacter* genus, all produced the same signalling molecule OC10-HSL.

### 3.3. Ecological Functions of the QS Bacteria

#### 3.3.1. Film-Forming Ability of the QS Bacteria

**Figure 3:**
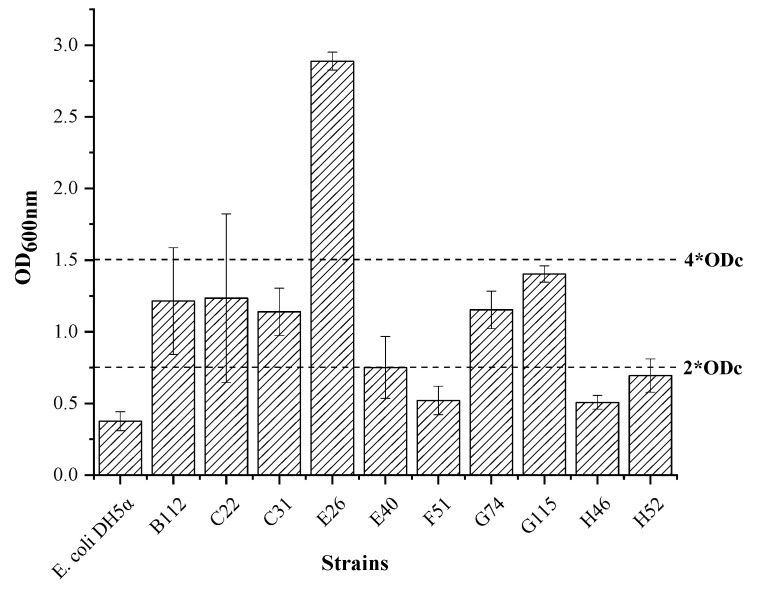
The film forming ability of QS bacteria. *E. coli* DH5α was the negative control and its OD_600nm_ was presented as the ODc. The line-2*ODc was twice ODc. The line-4*ODc was four times ODc.

The results of the 10 QS bacterial biofilm formation abilities showed that strains E40, F51, H46, and H52 had weak biofilm formation capabilities with OD values of 0.751, 0.520, 0.507 and 0.694, respectively (Figure 3). Strains B112, C22, C31, G74 and G115 had medium biofilm formation ability with OD values of 1.214, 1.234, 1.139, 1.152 and 1.403, respectively. Strain E26 had a strong biofilm formation ability with an OD value of 2.887. The strains that produced more types of signalling molecules had a stronger film-forming ability.

**Figure 4:**
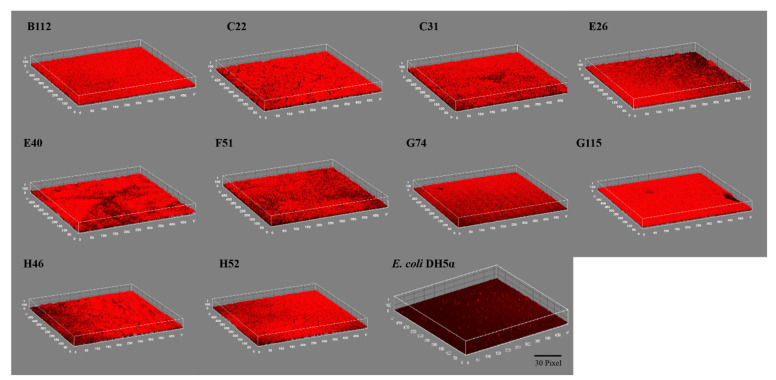
Confocal laser scanning microscope (CLSM) photomicrographs of 10 QS bacteria biofilms grown in 2216E liquid medium. All photomicrographs were taken at 72 h. *E. coli* DH5a was the negative control. Dyes of propidium iodide, wavelength of 559 and 570–670 nm and ×20 magnification were used to observe. Scale bar was 30 Pixel.

Since biofilm formation is a dynamic process, the biofilm morphology of 10 QS bacteria was observed by CLSM after three days of cultivation [38]. In this study, we selected the biomass, average thickness, average diffusion distance, and surface area to volume ratio of the microbial envelope to characterize the biofilm structure produced by these 10 QS bacteria. Biomass was measured as the number of microorganisms per unit area, the average thickness indicated the spatial size of the biofilm, and the average diffusion distance and the surface area to volume ratio reflected the proportion of the microbial biofilm that contacted the nutrient source and the ability of microbial coatings to be adapted to the environment [39]. In addition, we used COMSTAT software to analyse the various parameters of the microbial coating, and the results are presented in Figure 4.

After three days of cultivation, the microbial coating growth of the 10 QS bacteria is shown in Figure 4. According to the intuitive judgement of the picture results, it could be concluded that the biofilm biomass of the 10 QS strains increased significantly and showed structured microbial envelope morphology. Among them, the biomass of C31, G115, H52 and G74 belonging to the *Ponticoccus* genus was larger, 6.634–8.686 mg/cm^3^. Due to the rich nutrients in the 24-well plate, each strain grew rapidly, and the biofilm could cover the entire substrate surface with an average thickness of 10.637–19.710 μm. However, it also showed that the growth rate of the 10 QS strains was not uniform. Among them, strain G115, which had a relatively strong film forming ability, had the largest average thickness, which reached 19.710 μm. Strain E26, which had the strongest film-forming ability, also had a larger average thickness of 19.129 μm.

#### 3.3.2. Utilization of DMSP by QS Bacteria

**Figure 5:**
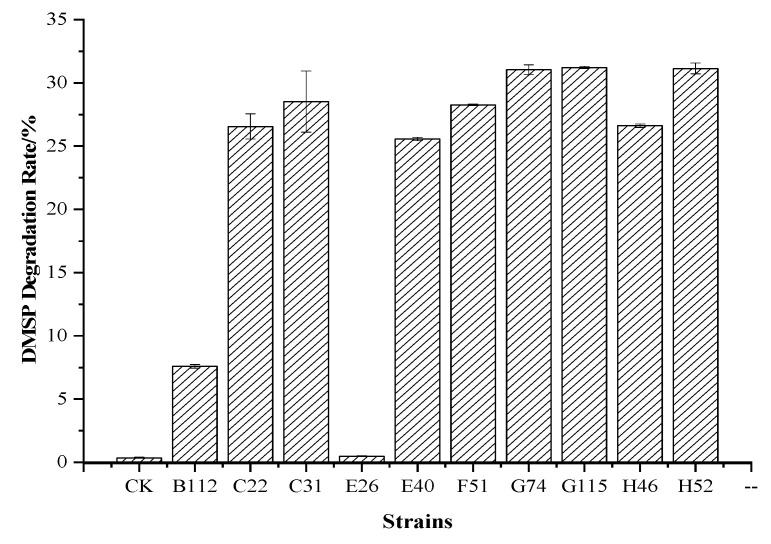
DMSP degradation by the QS bacteria. DMSP degradation was taken at 12 h by headspace gas chromatography (GC). CK was the blank control.

Alkaline hydrolysed into DMS was used to determine the degradation rate of DMSP. As shown in Figure 5, compared with the blank control group, most of the QS bacteria could degrade DMSP. The G74, G115 and H52 strains degraded DMSP in the medium at 31.037%, 31.218%, and 31.144%, respectively, showing strong DMSP degradation ability. The degradation of DMSP by the B112, C22, C31, E40, F51 and H46 strains was 7.585%, 26.552%, 28.518%, 25.564%, 28.263%6 and 26.609%, respectively. In addition, the E26 strain did not have the ability to degrade DMSP.

#### 3.3.3. The Influence of QS Bacteria on the Growth of Microalgae

**Figure 6:**
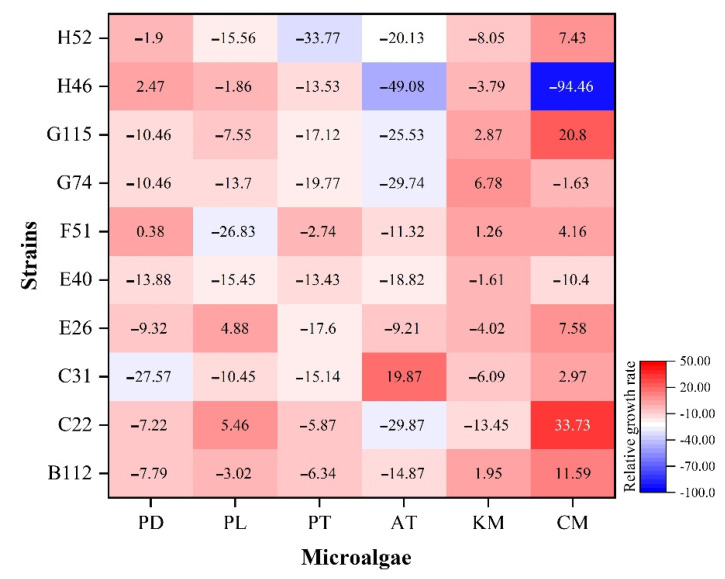
The effect of QS bacterial metabolites on the growth of microalgae.

The crude extracts of QS bacteria had different effects on the growth of the microalgae (Figure 6). The crude metabolic extract of the H52 strain had a strong inhibitory effect on *P. tricornutum*, and the inhibitory rate was 33.77%. The crude metabolic extract of the H46 strain had a strong inhibitory effect on *A. tamarense* and *C. marina*, with inhibition rates of 49.08% and 94.46%, respectively. At the same time, the experimental results also showed that the crude metabolic extracts of the B112, C22, and G115 strains could promote the growth of *C. marina*, and the promotion effect could reach 11.59%, 33.73%, and 20.80%, respectively. The crude extract of the C31 strain also had a promoting effect on *A. tamarense*, with a promotion rate of 19.87%. The crude metabolic extracts of the other strains did not significantly inhibit or promote the marine red tide algae.

## 4. Discussion

In this study, 113 QS bacteria were screened from 842 cultivable microalgae-associated strains in the phycosphere. After removing the repetitive QS bacteria, we selected 10 different bacterial strains that produced stable AHLs for phylogenetic analysis. The results showed that most of the QS bacteria belonged to the *Roseobacter* clade (Figure 1). Among them, *Ponticoccus* (6 strains) was the dominated genus (Table 1). The members in the *Roseobacter* clade of α-Proteobacteria are among the most abundant bacteria in the phycosphere, accounting for about 15–50% of the entire planktonic bacterial community [40,41]. It had been reported that the *Roseobacter* clade occupy an essential ecological position in the relationship between algae and bacteria. The *Roseobacter* clade had some ecological function, such as biofilm formation, DMSP degradation, and algicidal character affecting algae-bacterial interactions [31]. There was evidence that 80% of the *Roseobacter* clade can produce AHLs signalling molecules [42]. According to the above data, the proportion of QS bacteria in the phycosphere should be relatively high, about 12–40%. Our results also confirmed that in the initial screening process, QS bacteria accounted for 13.42% of the total culturable microalgae-associated bacteria, significantly higher than Lv’s results of 6.7% [30].

It was reported that 59% α-Proteobacteria can produce long side-chain AHLs (C10-HSL, C13-HSL to C16-HSL, C18-HSL) in marine environment [43]. Long side-chain AHLs with chain lengths of C14-HSL, C16-HSL and C18-HSL obviously dominated among the identified compounds [43]. The results showed that bacteria form biofilms on the surface of the organisms (marine plants and animals) regulated by QS [43]. Since they tend to be retained in the cells, long side-chain AHLs seem less susceptible to hydrolysis caused by pH fluctuations (due to photosynthesis) in marine biofilms [44]. In our study, we found that QS bacteria produce various AHLs signalling molecules such as C6-HSL, C8-HSL, OC8-HSL, OC10-HSL, and 4 unidentified AHLs (short side-chain AHLs: N3, long side-chain AHLs: N1, N2, N4) (Figure 2). Most of these AHLs signalling molecules are long side-chain AHLs and eight of these bacteria could produce long-chain AHLs. Due to the limited types of AHL standards, AHLs of N1-N4 need to be identified further. Although different bacterial species produce the same AHLs signalling molecules, they may have different roles in regulating functional genes and phenotypes [40]. Rivas et al. confirmed that the C6-HSL generated by *Pseudomonas* sp. and *Rhizobium* sp. can significantly increase the biomass of *Botryococcus braunii* [45]. However, Bi et al. found that under 400 nmol/L C6-HSL stress, the photosynthesis of *Chlorella vulgaris* PSII was inhibited, and its anaerobic respiration rate was enhanced [46].

Evidence was found that the QS system regulate divers ecological functions. We believe QS in phycosphere are likely to regulate bacterial behaviours that are crucial for bacteria-phytoplankton interaction [47]. In our study, all the ten strains were observed have film-forming abilities by the crystal violet staining method (Figure 3) and CLSM method (Figure 4). COMSTAT analysis results showed that the average diffusion distance of membranes was relatively low, while the ratio of surface area: volume showing a negative correlation trend was relatively large (Table 2). This indicated that in the stage of microbial film formation, bacteria randomly attached to the surface of the substrate. A previous study has showed that the formation of a microbial biofilm is regulated by various chemical signals, the most important of which is the AHLs signalling molecules [48]. Whiteley et al. detected gene expression in the membrane of *Pseudomonas aeruginosa* by the microarray method and found that approximately 12% of the activated or repressed genes were related to QS [49]. The formation of biofilm promoted the algae-bacterial interactions, including mutual benefit, symbiosis and antagonism [50,51,52,53]. We believe that QS bacteria are easier to form biofilm in phycosphere, thus influencing some ecological functions probably, such as DMSP degradation, algicidal character.

Current research shows that marine microalgae are the primary producers of DMSP, and marine bacteria are the principal decomposers of DMSP. Studies have reported that the occurrence of red tides and microalgae cultivation is accompanied by apparent degradation of DMSP by marine bacteria, such as *Phaeocystis* sp., *Heterocapsa triquetra*, *Scrippsiella trochoidea* and other microalgae. Marine bacteria degrade DMSP through demethylation pathways and lysis pathways [54]. Among them, lysis metabolism can generate DMS. The resulting DMS accounts for more than 90% of the total output of marine DMS and is the primary source of DMS in the world [54]. Studies have also found that the algal bacterial communities of many marine algae are rich in bacteria with DMSP degradation functions. The known DMSP lytic bacteria mainly come from the α-Proteobacteria (Alphaproteobacteria) and γ-Proteobacteria (Gammaproteobacteria), of which the *Roseobacter* clade and the SAR11 clad are the most critical groups for cracking DMSP [55]. Moreover, DMSP is the preferred source of reducing sulfur for *Rhodobacter* clade bacteria, even though its concentration is 10 times lower than that of sulfate in seawater [56]. Studies have found that 12 species of bacteria in the *Roseobacter* cluster can degrade and metabolize sulfides, including *Sulfitobacter* and *Leisingera* [40]. In this study, C22, E40 and H46 which belong to *Sultiobacter* and *Leisingera* can degrade DMSP. Johnson et al. found that DMSP produced by phytoplankton cells can further trigger the production of the QS signalling molecules in *Roseovarius pomeroyi* [57]. Then a series of metabolic changes occur, such as increasing the uptake of dissolved organic nitrogen (DON) and releasing sulphur metabolites such as MeSH, DMS and 5′-deoxy-5′-methylthioadenosine (MTA) [58]. We believe that this kind of microalgae-associated QS bacteria with DMSP degradation ability plays a vital role in regulating the growth and elimination of microalgae. They can indirectly regulate the biomass of microalgae. When algae biomass increases, the concentration of DMSP also increases, and QS bacteria degrade DMSP to produce DMS. DMS has a particular regulatory effect on regional climate change [59]. An increase in DMS is conducive to the formation of clouds to block sunlight, weaken the photosynthesis of algae, and indirectly reduce the growth rate of microalgae, which may also be a way to maintain a balance of algal microecology.

Competition between algae and bacteria is common because of food and space constraints. Bacteria use a variety of strategies to obtain resources, including secreting toxins and producing harmful compounds against algae or other organisms that affect their life cycle. Our results showed that most of the QS bacteria’ crude metabolites inhibit the growth of microalgae. In this study, 27 groups had inhibitory effect on the microalgae in the 60 groups (more than 10% inhibition rate) (Figure 6). It has been reported that the metabolites of QS bacteria have inhibitory effect on the microalgae. Nakashima et al. confirmed that PG-L-1 pigment produced by MS-02–063 strain was controlled by QS, showing potent algicidal activity against various red tide phytoplanktons [60]. Guo et al. verified that two algicidal compounds (3-benzyl-piperazine-2,5-dione and 3-methylindole) produced by *Aeromonas* sp. strain GLY-2107 were controlled by C4-HSL, showing high algicidal activities against *Microcystis aeruginosa* [61]. We believe that the crude metabolite extract of the microalgae-associated QS bacteria contain algicidal compounds. Through the anti-algae experiment of QS bacteria, it was found that the anti-algae activity of QS bacteria has pronounced species specificity. In this experiment, the crude metabolite extract of the H46 strain had a strong inhibitory effect on *A. tamarense* and *C. marina*, with inhibition rates reaching 49.08% and 94.46%, respectively, but not significantly for the other algae. We also found that some crude metabolites of QS bacteria promote the growth of algae. The crude metabolic extracts of B112, C22, C31, and G115 strains can promote the growth of *C. marina* and *A. tamarense* (11.61%–33.93%). During HAB, bacteria provide some of the necessary nutrients and resources for toxin-producing microalgae. Since bacterial population density is regulated by the QS system, developing QS inhibitors that block QS biological functions may be a way to control algal concentrations and limit the effects of their toxins [62]. The application of QS inhibitors to limit HAB growth has been reported. For example, using ethyl 2-methylacetoacetate (EMA) as a signalling inhibitor, Hong successfully destroyed the balance of cellular redox processes in *Microcystis aeruginosa*, thereby limiting its growth [63]. *Phaeobacterium gallaeciensis* (Rhodobacterales) can establish a dynamic interaction relationship with *Emiliana huxleyi*. At the initial stage of coculture, *P. gallaeciensis* can promote the growth of *E. huxleyi*, but when *E. huxleyi* enters the declining stage, *P. gallaeciensis* begins to secrete alginolytic substances to dissolve and rupture *E. huxleyi*. This shows that algae and algal bacteria do not simply provide nutrients, vitamins, iron ions, etc., but they have more complex interactions. Therefore, it is necessary to study the combined evolutionary process of microalgae, bacteria and QS signals, which is conducive to elucidating the potential mechanism of how bacterial behaviour affects the formation and development of HAB.

## 5. Conclusions

The species of QS bacteria, AHL signalling molecules, and ecological functions of HAB microalgae-associated microorganisms were studied. We found that most of the QS bacteria belong to the *Roseobacter* cluster and have the capacity to form biofilms and degrade DMSP. Studying the influence of QS bacterial metabolites on the growth of microalgae showed that most of the metabolites of QS bacteria inhibited the growth of microalgae. We speculate that the possible ecological function of QS bacteria in the phycosphere is to form biofilms on the surface of the microalgae to facilitate their utilization of the crucial organic matter produced by the microalgae and the DMSP, and release some algae inhibitory substances into the surrounding environment, thereby controlling the microalgae. Experiments to test these hypotheses are under way in our laboratory.

## Figures and Tables

**Table 1:** Identification of 16S rRNA sequences of 10 QS bacteria isolated from typical marine red tide algae.

Strain (Accession Number)	Closest Match in GenBank (Accession Number)	Similarity (%)	Alignment Fragment Size (bp)
B112 (MN868699)	*Ponticoccus alexandrii* strain AT2-A (KY012361.2)	99	1335
C22 (MN868700)	*Sulfitobacter* sp. strain QD214-NT-105 (JQ675546.1)	99	1319
C31 (MN868701)	*Ponticoccus* sp. strain 7002-105 (KY770533.1)	99	1321
E26 (MN868702)	*Nitratireductor* sp. strain L16S-12 (KY770526.1)	99	1365
E40 (MN868703)	*Leisingera caerulea* strain DSM 24564 (NR_118542.1)	99	1309
F51 (M868704)	*Ponticoccus* sp. LZ-7 (MG704132.1)	99	1318
G74 (MN868705)	*Ponticoccus* sp. strain 7002-105 (KY770533.1)	99	1305
G115 (MN868706)	*Ponticoccus* sp. strain 7002-105 (KY770533.1)	99	1319
H46 (MN868707)	*Sulfitobacter* sp. strain KMM6719 (KC2247329.1)	100	1319
H52 (MN868708)	*Ponticoccus* sp. strain 7002-105 (KY770533.1)	99	1345

**Table 2:** Biofilm analysis results of QS bacteria based on COMSTAT analysis software.

	Biomass (mg/cm^3^)	The Average Thickness (μm)	Average Diffusion Distance	Surface Area to Volume Ratio (μm^2^/μm^3^)
B112	7.311 ± 0.195	18.687 ± 0.407	0.064 ± 0.001	2.722 ± 0.02
C22	5.918 ± 0.344	16.210 ± 0.920	0.068 ± 0.003	2.430 ± 0.020
C31	7.877 ± 0.090	20.785 ± 1.454	0.053 ± 0.003	2.369 ± 0.092
E26	7.067 ± 0.219	22.132 ± 0.277	0.046 ± 0.002	2.110 ± 0.093
E40	5.274 ± 0.168	14.134 ± 0.904	0.048 ± 0.004	2.285 ± 0.076
F51	5.367 ± 0.715	15.874 ± 2.107	0.059 ± 0.006	2.622 ± 0.044
G115	8.686 ± 0.390	23.928 ± 1.255	0.102 ± 0.008	1.712 ± 0.110
G74	6.634 ± 1.570	18.707 ± 4.686	0.061 ± 0.018	2.622 ± 0.026
H46	5.142 ± 0.713	13.157 ± 1.710	0.110 ± 0.013	2.268 ± 0.043
H52	7.844 ± 0.720	21.793 ± 2.626	0.048 ± 0.007	2.323 ± 0.014
*E. coli* DH5α	0.429 ± 0.547	0.2594 ± 0.636	0.012 ± 0.824	4.386 ± 0.049

## Data Availability

Data are available upon request; please contact the contributing authors.
